# Novel Insight into the Role of Squalene Epoxidase (*SQLE*) Gene in Determining Milk Production Traits in Buffalo

**DOI:** 10.3390/ijms24032436

**Published:** 2023-01-26

**Authors:** Chao Chen, Xiangwei Hu, Muhammad Jamil Ahmad, Kaifeng Niu, Tingzhu Ye, Aixin Liang, Liguo Yang

**Affiliations:** 1 National Center for International Research on Animal Genetics, Breeding and Reproduction (NCIRAGBR), College of Animal Science and Technology, Huazhong Agricultural University, Wuhan 430070, China; 2 Hubei Province’s Engineering Research Center in Buffalo Breeding and Products, Wuhan 430070, China

**Keywords:** *SQLE*, buffalo, mammary epithelial cells, proliferation, apoptosis, association analysis

## Abstract

Understanding the genetic mechanisms underlying milk production traits contribute to improving the production potential of dairy animals. Squalene epoxidase (*SQLE*) is one of the rate-limiting enzymes for cholesterol biosynthesis and was highly expressed in the buffalo mammary. The objectives of the present study were to detect the polymorphisms within *SQLE* in buffalo, the genetic effects of these mutations on milk production traits, and to understand the gene regulatory effects on buffalo mammary epithelial cells (BuMECs). A total of five SNPs were identified by sequencing, g.18858G > A loci were significantly associated with fat yield, and g.22834C > T loci were significantly associated with peak milk yield, milk yield, fat yield, and protein yield. Notably, linkage disequilibrium analysis indicated that 2 SNPs (g.18858G > A and g.22834C > T) formed one haplotype block, which was found to be significantly associated with milk fat yield, fat percentage, and protein yield. Furthermore, expression of *SQLE* was measured in different tissues of buffalo and was found to be higher in the mammary. Knockdown of *SQLE* gene expression significantly affected the growth of BuMECs, including proliferation, cell cycle, and apoptosis, and significantly downregulated the expression of related genes *MYC*, *PCNA*, and *P21*. In addition, knockdown of the *SQLE* gene significantly reduces triglyceride concentrations and the signal intensity of oil red O staining. In addition, silencing of *SQLE* was also found to regulate the synthesis and secretion of β-casein and κ-casein negatively. Furthermore, *SQLE* knockdown is accompanied by the downregulation of critical genes (*RPS6KB1*, *JAK2*, *eIF4E*, and *SREBP1*) related to milk fat and protein synthesis. The current study showed the potential of the *SQLE* gene as a candidate for buffalo milk production traits. It provides a new understanding of the physiological mechanisms underlying buffalo milk production regulation.

## 1. Introduction

Buffalo milk represents an indispensable source of nourishment in many parts of the world and it is the second most consumed milk worldwide [1,2]. Buffalo has important economic and biological significance for tropical and subtropical regions due to characteristics of good adaptability and stress resistance [3]. So, livestock herders in Pakistan consider buffalo as a “black gold” [4]. Buffalo milk has a higher nutritional value in fat, protein, and iron, and less cholesterol than dairy cow milk [5]. The high-fat content of buffalo milk makes it also highly suitable for processing, and in developed countries it is mainly used for the production of a variety of foodstuffs, such as butter oil, cheeses, and ice cream. In addition, the highly popular buffalo Mozzarella is obtained under the European Union’s protected designation of origin scheme in Italy [6,7]. However, the low milk yield has been limiting the buffalo industry’s progress. Therefore, improving the buffalo milk yield while maintaining its high milk quality is the major challenge for modern buffalo breeding.

The continued advancement of high-throughput genotyping and sequencing technologies allowed the discovery of hundreds of thousands of genetic markers in the genomes of animals, such as single-nucleotide polymorphisms (SNPs) [8]. Genomic selection mainly depends on the linkage disequilibrium (LD) among genetic markers and loci associated with the trait variation to create the prediction equation [9,10]. Candidate gene approaches and genome-wide association studies can be used to identify these genetic loci [11]. The candidate gene approach has been established as an effective way to choose the candidate genes affecting milk production traits [12,13].

Squalene epoxidase (*SQLE*) is a microsomal flavin monooxygenase that catalyzes the first oxygenation step in cholesterol synthesis, the conversion of squalene into the nonsterol precursor for lanosterol, 2,3-(S)-monooxidosqualene (MOS), and is one of the rate-limiting enzymes for cholesterol biosynthesis [14,15,16]. Recently, studies have reported that the SNP in the *SQLE* gene was associated with several meat quality traits including back-fat thickness, carcass weight, meat color (yellowness), fat composition, and water-holding capacity [17]. Subsequently, knockdown of *SQLE* expression in 3T3-L1 pre-adipocytes was found to significantly inhibit adipogenesis [17]. In addition, previous studies have demonstrated the association of SQLE with cholesterol metabolism and adipogenesis [18,19,20].

As the individual mammary gland matures, the epithelial cells of the mammary gland begin to develop under hormonal control and participate in the production and secretion of milk [21,22]. It has been found that apoptosis and a decrease in mammary epithelial cells during lactation lead to a decrease in lactation [23]. So, the study of mammary epithelial cells is essential to improve milk quality and milk production. Numerous studies have established that *SQLE* genes are involved in the proliferation and migration of many types of cancer cells (lung squamous cell carcinoma cells and hepatocellular carcinoma cells) [24,25,26]. Moreover, SQLE copy number has been reported to decrease cell viability and increase replication time in breast cancer cell lines [27].

Therefore, the objectives of this study were to identify genetic mutations of the *SQLE* gene in buffalo, and detect the association between these genetic markers and milk production traits in the tested populations. Furthermore, we tried to explore the regulatory role of *SQLE* on buffalo mammary epithelial cells (BuMECs) proliferation, triglycerides, and lipid distribution, as well as casein synthesis.

## 2. Results

### 2.1. SNP Polymorphic Loci Identification and Genotyping of SQLE Gene

**Figure 1:**
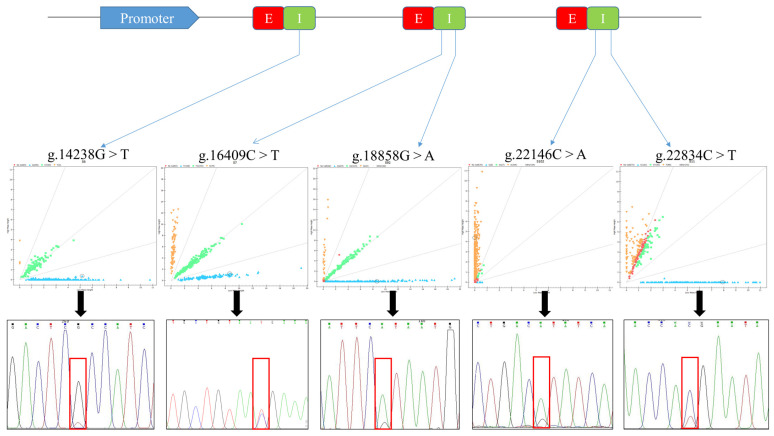
Schematic representation of buffalo *SQLE* gene with the localization of five identified SNPs and their SNP genotyping plots. E: exon region; I: intron region. Red squares represent mutant loci, different colored lines represent different bases.

Five potential SNPs (g.14238G > T, g.16409C > T, g.18858G > A, g.22146C > A, and g.22834C > T) were identified in the buffalo DNA pool sequencing data from the tested samples (Figure 1). All SNPs are located within the intron region, g.14238G > T in the sixth, g.16409T > C and g.18858A > G in the seventh, g.22146A > C and g.22834T > C in the ninth. MALDI-TOF-MS genotyping detected SNPs in 331 of the Italian Mediterranean buffalo sample. The genetic diversity of the studied population for these five SNPs was discovered by estimating the genotype frequencies, allele frequencies, polymorphism information content (PIC), and heterozygosity (Table 1).

### 2.2. Association of SQLE Genotype with Milk Production Traits

To know whether identified SNPs of the *SQLE* gene in Italian Mediterranean buffaloes are associated with milk production traits (peak milk yield, milk yield, fat yield, fat percentage, protein yield, and protein percentage), an association analysis was performed using a Mixed Linear Model and results were given (Table 2). The SNPs at g.18858G > A loci were significantly associated with fat yield (FY), with higher FY in the AG genotype (235.45 ± 3.67 kg) compared to the AA genotype (225.47 ± 2.93 kg, *p* < 0.05). The SNPs at g.22834C > T had a significant association with the following milk production traits. Compared to the CC genotype, the TT genotype showed significant increases in peak milk yield (PM), milk yield (MY), and FY of 0.61 kg, 130.67 kg, and 13.06 kg, respectively (*p* < 0.05). Additionally, there was a significantly higher protein yield (PY) in the TT genotype (134.53 ± 2.22 kg) compared to the CC genotype (128.07 ± 1.90 kg, *p* < 0.05). However, the SNPs at g.14238G > T, g.16409C > T and g.22146C > A loci were not significantly associated with buffalo milk production traits (*p* > 0.05).

### 2.3. Linkage Disequilibrium and Haplotypes Analysis

**Figure 2:**
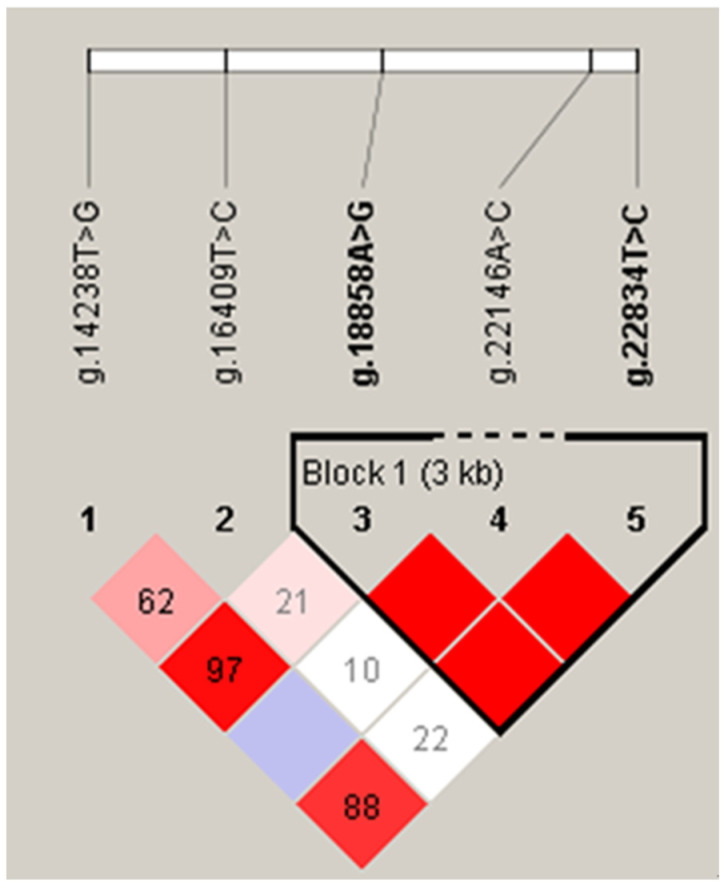
Linkage disequilibrium among the five SNPs of the SQLE gene. The “Block 1 (3 kb)” indicates haplotype block, and the text above the horizontal number is the SNP name. The red squares represent high pairwise linkage disequilibrium, coloring down to white squares of low pairwise linkage disequilibrium.

We further performed linkage disequilibrium (LD) and haplotype analysis for the five detected SNPs, and one haplotype block was identified (Figure 2). This haplotype block was composed of three haplotype combinations, H1H1: CA/CA, H1H3: CA/TG, and H2H3: TA/TG with frequencies of 56.50%, 20.24%, and 16.01%, respectively. In addition, we observed that individuals with the haplotype combination H1H1 (235.78 ± 3.44 kg) had significantly higher FY than those with H2H3 (225.02 ± 3.09 kg, *p* < 0.05). Compared to individuals with haplotype combination H1H3, individuals with haplotype combination H1H1 had a significant increase in FP of 0.30% (*p* < 0.05). Furthermore, individuals with haplotype combination H1H1 had significantly higher PY than individuals with haplotype combination H2H3 (*p* < 0.05). However, different haplotype combinations had no significant effect on PM, MY, and PP (*p* > 0.05, Table 3).

### 2.4. Identification of BuMECs, Interference Efficiency, and Expression Profile of SQLE

**Figure 3:**
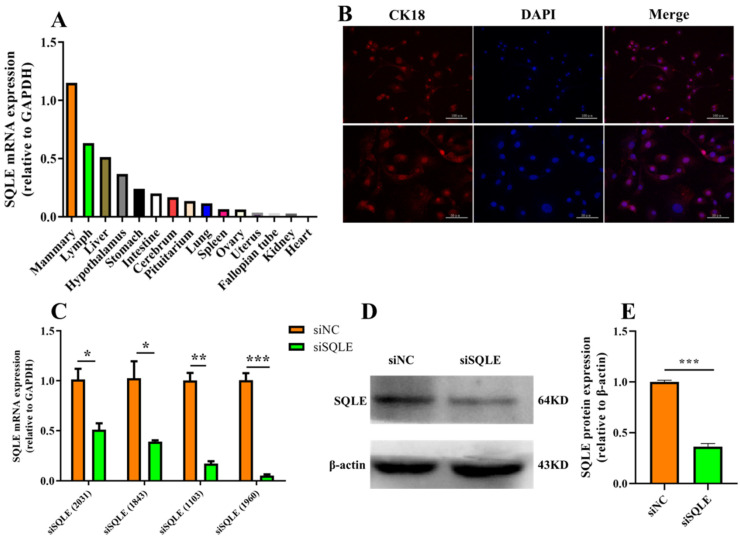
*SQLE* gene expression profile and interference efficiency. (**A**) mRNA expression profile of SQLE in different tissues of buffalo by quantitative real-time PCR method; (**B**) immunofluorescence identification of BuMECs with cytokeratin 18; (**C**) BuMECs were transfected with siSQLE, and fluorescence quantitative qPCR was used to detect the *SQLE* mRNA levels; (**D**,**E**) Western Blot detected the protein expression of *SQLE*. *n* = 3 biological replicates. * *p* < 0.05, ** *p* < 0.01, *** *p* < 0.001, compared with control group. Error bars represent SEM.

The *SQLE* gene was found to be constitutively expressed at different levels in all investigated tissues (mammary, lymph, liver, hypothalamus, stomach, intestine, cerebrum, pituitarium, lung, spleen, ovary, uterus, fallopian tube, kidney, and heart) in buffalo. The mRNA expression of *SQLE* was highest in the mammary and lowest in heart (Figure 3A). Meanwhile, the isolated BuMECs were identified using immunofluorescence detection of cytokeratin 18 (*CK18*), a marker protein of epithelial cells (Figure 3B) [28]. To uncover the biological function of *SQLE* in BuMECs, four different siRNAs were designed and synthesized: siSQLE (2031), siSQLE (1843), siSQLE (1103), and siSQLE (1960). The knockdown efficiency of *SQLE* mRNA level by siSQLE (1960) transfection reached 95.19%, compared to control siRNA-transfected cells (*p* < 0.001, Figure 3C). We selected siSQLE (1960) for subsequent experiments because it was the most effective siRNA. In addition, compared with the siNC group, the *SQLE* protein relative expression of the siSQLE group was significantly lower (*p* < 0.001, Figure 3D,E).

### 2.5. SQLE Regulates the BuMECs Proliferation, Cell Cycle, and Apoptosis

**Figure 4:**
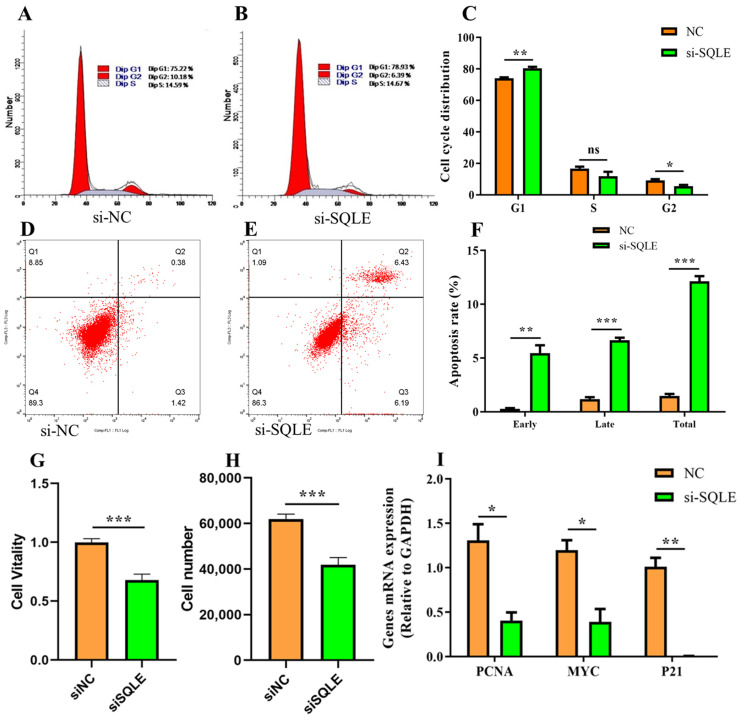
Effect of knockdown *SQLE* gene on BuMECs proliferation and apoptosis. (**A**) (si-NC) and (**B**) (si-SQLE) cell cycle distribution; (**C**) flow cytometry was used to detect cell cycle progression; (**D**) (si-NC) and (**E**) (si-SQLE) cell apoptosis distribution; (**F**) the rates of early, late, and total apoptosis were assessed. (**G**) The CCK-8 assay showed that *SQLE* knockdown resulted in a significant decrease in cell viability; (**H**) the cell counting test showed that BuMECs were significantly decreased after *SQLE* knockdown; (**I**) mRNA expression of cell cycle, proliferation, and apoptosis–related genes. *n* = 3 biological replicates. * *p* < 0.05, ** *p* < 0.01, *** *p* < 0.001, ns: nonsignificant difference, compared with control group. Error bars represent SEM.

Flow cytometry analysis for the cell cycle indicated that the cell cycle was arrested in siSQLE-treated cells compared to the control, as there was a significant decrease in the G2 (3.53%, *p* < 0.05) and increase in the G1 (6.42%, *p* < 0.05) phase (Figure 4A–C). Furthermore, the apoptosis rate was determined by transfecting BuMECs for 48 h with siSQLE or siNC. The results showed that siSQLE-treated BuMECs had a significantly higher early (5.47 ± 0.72), late (6.66 ± 0.23), and total apoptosis rate (12.13 ± 0.49) compared to the siNC (early apoptosis rate: 0.29 ± 0.09; late apoptosis rate: 1.19 ± 0.25; total apoptosis rate: 1.48 ± 0.26), respectively (*p* < 0.01, Figure 4D–F). Meanwhile, to confirm the *SQLE* role in cell proliferation, we performed CCK-8 assays to examine the effect of *SQLE* on the viability of BuMECs. The results demonstrated that cell viability was significantly reduced by *SQLE* knockdown (*p* < 0.001, Figure 4G). In addition, the cell counts were measured with an automatic cell counter, and the results revealed a significant decrease in the number of cells in *SQLE* knockdown cells (*p* < 0.001, Figure 4H). Consistently, *SQLE* gene knockdown inhibits the cell cycle, proliferation, and apoptosis-related gene (*PCNA*, *MYC*, and *P21*) expression (*p* < 0.05, Figure 4I).

### 2.6. SQLE Regulates BuMECs Lipogenesis and Casein Synthesis

**Figure 5:**
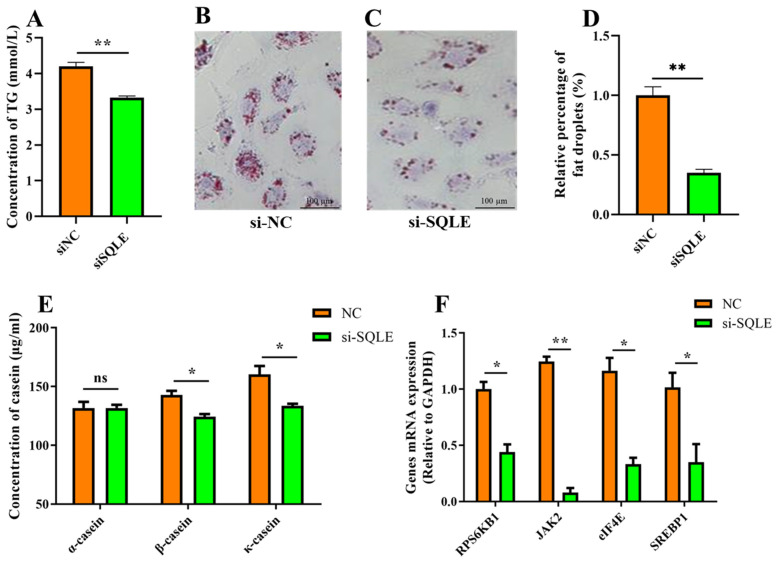
*SQLE* regulate lipogenesis and casein synthesis in BuMECs. (**A**) Triglyceride concentration was detected in the cell lysate. Triglyceride concentration was normalized by control; (**B**–**D**) Oil red O staining was used to indicate the lipid droplet secretion of BufMECs (Scare bars: 100 μm); (**E**) content of α-casein, β-casein, and κ-casein in the BuMECs culture supernatant was determined by ELISA. (**F**) mRNA expression of milk fat and protein synthesis-related genes after *SQLE* knockdown, and *GAPDH* was used as the inner control. *n* = 3 biological replicates. * *p* < 0.05, ** *p* < 0.01, ns: nonsignificant difference, compared with control group. Error bars represent SEM.

To make a thorough exploration of *SQLE* function in mammary epithelial cells, we detected the effect of *SQLE* on milk fat and milk protein synthesis. The secretory effect of *SQLE* on the triglyceride (TG) level, a major lipid milk fat, was examined. The results showed that *SQLE* knockdown significantly reduced the secretion of TG from BuMECs (*p* < 0.01, Figure 5A). The oil red O staining of neutral lipid accumulation confirms the reduction of lipid droplets in *SQLE* knockdown cells (*p* < 0.01, Figure 5B–D). Our previous association study revealed that *SQLE* mutation affected the milk protein yield (Table 2). We further performed the ELISA assay to detect the α-casein, β-casein, and κ-casein levels in the culture medium of the BuMECs after *SQLE* silencing. The results showed that the concentrations of β-casein and κ-casein were significantly lower in siSQLE-treated BuMECs culture medium compared to siNC (*p* < 0.05), but there was no significant difference in α-casein (*p* > 0.05, Figure 5E). Furthermore, we examined the expression of genes related to milk fat and protein synthesis in siSQLE-treated BuMECs. Consistently, *SQLE* gene knockdown significantly inhibits the expression of the milk fat and protein synthesis-related genes *RPS6KB1*, *JAK2*, *eIF4E*, and *SREBP1* (*p* < 0.05, Figure 5F).

## 3. Discussion

Milk yield and quality have been the primary selection criteria for genetic improvements in livestock species, which are complex quantitative traits regulated by different genes [29]. Thus, identifying candidate genes associated with milk production traits may provide information that can be used to enhance the accuracy of animal selection for moderately heritable traits, such as milk production [30]. In addition, 19 candidate genes associated with milk production traits in buffalo were reported, including *ADRA1A*, *A2M*, *BTN1A1*, *DGAT1*, *CSN1S1*, *GHRL*, *INSIG2*, *LALBA*, *LEP*, *MC4R*, *MTNR1A*, *OXT*, *PRL*, *SCD*, *SPP1*, *SREBF1*, *STAT1*, *STAT5A,* and *TG* genes [11]. Previous studies have shown that SNPs in the *SQLE* gene are significantly associated with several meat quality traits in pigs [17]. However, no literature has been reported on SNPs in the *SQLE* gene affecting milk production traits in buffalo. Milk production is a polygenic trait, regulated by several genes [31,32]. Thus, animal producers desire to know the genetic changes encoding the preferred phenotypes.

In our study, the associations of SNPs in the *SQLE* gene with milk production traits in buffaloes were analyzed for the first time. Although the SNPs identified in the current study were all located in the intron region, some studies have reported that introns can affect gene transcription initiation and elongation, mRNA transport out of the nucleus, mRNA stability, and ultimately mRNA translation efficiency [33,34]. Our results show that SNPs in the *SQLE* gene have a significant effect on the buffalo milk fat and protein yield. In addition, we found a haplotype block formed by the SNPs identified in the *SQLE* gene. A “haplotype” refers to a group of alleles inherited on a single chromosome containing DNA sequences with multiple SNPs [35,36]. Haplotype can distinguish the genetic information of different parental chromosomes, and reveals the combination and inheritance of different genetic sites on a single chromosome or a specific single chromosome region, which is helpful to explore the heterosis in animals [37]. Our results demonstrate that this haplotype block significantly affects fat yield, fat percentage, and protein yield in buffalo milk. It can be inferred that the *SQLE* gene may play an essential role in milk lipid metabolism and protein synthesis in buffalo.

To further reveal the effects of the *SQLE* gene on milk lipid metabolism and protein synthesis in buffalo milk, we examined the impacts of knockdown of the *SQLE* gene on BuMECs, including proliferation, apoptosis, cell cycle, and milk fat and milk protein synthesis. It is well known that the mammary gland synthesizes and secretes milk composition. Mammary epithelial cells (MECs) are an important component of the mammary gland. Most milk compositions, including milk protein and milk fat, are synthesized and secreted by MECs [38], which are widely used over the recent years as a model to understand the physiological function of the mammary gland, which offers an effective tool for the study of cell proliferation, apoptosis, lipogenesis, and casein synthesis [39,40]. The current siSQLE-induced arrest of cell cycle with an increase in G1 phase and a decrease in G2 phase of BuMECs is consistent with He et al. and You et al.’s previous studies that silencing the expression of the *SQLE* gene can inhibit the proliferation of tumor cells [41,42]. However, the effect of *SQLE* genes on the proliferation of BuMECs has not been reported. To further elucidate the mechanism of *SQLE* gene regulation of BuMECs proliferation, we examined the expression of proliferation-related genes *PCNA*, *P21*, and *MYC*, and the results showed that they were significantly downregulated (*p* < 0.05). Numerous studies have demonstrated that the absence of *MYC* inhibits cell growth [43,44,45]. In addition, Ushmorov, A., K.M. Debatin et al. found that overexpression of *MYC* leads to an increase in G2 stage cells [46]. Our results show that *MYC* was downregulated and significantly decreased in G2 phase cells, which is consistent. *PCNA* is crucially regulated by the tumor suppressor protein p21 (*WAF1*) [47], which was initially identified as a potent inhibitor of cell-division cycle kinases (CDKs). *PCNA* binding is mediated by a PIP box located in p21’s C-terminal tail [48,49]. *PCNA* downregulation is demonstrated to inhibit cell proliferation [50], and *P21* downregulation can promote apoptosis [51]. In the present study, our data indicated that *SQLE* knockdown significantly (*p* < 0.05) inhibited the expression of *PCNA*, *MYC*, and *P21*, which further supported that *SQLE* might regulate BuMECs proliferation and apoptosis. Moreover, previous studies reported that the quantity and milk performance of MECs determined the milk production and quality of bovines [52]. This suggested that the role of *SQLE* to promote cell growth might be positively correlated with increased milk production.

More than 95% of milk fat is composed of triglycerides (TGs), which are synthesized in the endoplasmic reticulum and play a key role in the taste of milk, and are one of the most important factors in determining milk quality [53]. Previous study results showed that lipid droplet accumulation in adipocytes was significantly decreased by *SQLE* knockdown [17]. Overexpression of *SQLE* significantly increases TG levels in chicken hepatoma cells [54]. In the current study, oil Red O results show a significant reduction in lipid droplet aggregation in BuMECs after knockdown of *SQLE* gene expression (*p* < 0.01). Moreover, silencing of *SQLE* leads to a significant decrease in TG levels (*p* < 0.01). In these findings, *SQLE* may affect the milk fat rate of buffalo milk by influencing lipid synthesis in epithelial cells. *SREBP1* has been identified as a major transcription factor that activates genes regulating lipid and triglyceride synthesis, which can regulate the expression of milk fat synthesis-related enzymes and proteins, such as fatty acid binding protein 3 (*FABP3*), and proliferation of activated receptor gamma (*PPARγ*), thereby affecting the synthesis and transport of fatty acids [55,56]. Recent studies have demonstrated that silencing *SREBP1* expression inhibits the expression of milk fat synthesis-related genes in BuMECs [38]. In our study, *SQLE* knockdown significantly inhibited milk fat synthesis-related gene *SREBP1* mRNA expression (*p* < 0.01). Therefore, it is suggested that *SQLE* may affect milk fat secretion by affecting the *SREBP1* gene.

Casein content in milk is approximately 80% milk protein and mainly consists of α-, β-, and κ- casein, which are an important factors in determining milk quality [57,58]. In the present study, the knockdown of *SQLE* gene expression resulted in a significant decrease in the concentrations of β- and κ- casein, but no significant difference in the concentration of α-casein. PI3K-AKT-mTOR and JAK2-STAT5 pathways have been the major signaling pathways in milk protein synthesis [59]. mTOR regulates protein translation through its signaling pathway downstream of *4EBP1* and *RPS6K1* [60]. Activated *4EBP1* is phosphorylated and separated from *elF4E*, which binds to the translation initiation factor to initiate translation of the mRNA into a protein [61]. In the JAK2/STAT5 signaling pathway, activated *JAK2* phosphorylates *STAT5*, which activates the transcription of genes involved in the synthesis of milk proteins, thereby regulating the synthesis of milk proteins [62]. Our results show that knockdown of *SQLE* leads to significant downregulation of *elF4E*, *RPS6K1*, and *JAK2* expression in BuMECs. It was conjectured that *SQLE* may affect milk protein secretion by affecting JAK2-STAT5 and PI3K-AKT-mTOR pathways.

## 4. Materials and Methods

### 4.1. Samples Collection

A total of 331 buffalo blood DNA samples and relevant milk production records were derived from our previous studies [40,63]. All the milk production records (peak milk yield, milk yield, fat yield, fat percentage, protein yield, and protein percentage) were adjusted to 270 days [64]. The Italian Buffalo Breeders Association (ANASB) collected and provided the records. All experimental designs and animal treatment protocols were approved by the Ethical Animal Care and Use Committee of Federico II University of Naples (Italy), as previously described [65]. We extracted genomic DNA from the whole-blood samples of buffalo by using a kit method (QIAamp DNA Blood kit; Qiagen, Milan, Italy) following the instructions provided by the manufacturer. The Nanodrop 2000 spectrophotometer (Thermo-Fisher Scientific, Wilmington, DE, USA) and 1.5% agarose gel were used to determine the concentration and quality of extracted DNA.

### 4.2. SNP Identification, Genotyping, and Linkage Disequilibrium Analysis

Fifty buffalo samples were randomly selected to identify the variants of the *SQLE* gene by pooled DNA sequencing. A total of 12 pairs of SNP primers (Supplementary Table S1) based on the GenBank sequence (GenBank accession number: NC_059171.1) of the *SQLE* gene were designed (premier 5.0 software, Premier Biosoft, Palo Alto, CA, USA) and synthesized by Tsingke Biological Technology, Wuhan, China. PCR products were sequenced by the company (Tianyi Huiyuan, Inc, Wuhan, China), and the SeqMan software (DNASTAR Inc., Madison, WI, USA) was used to search the sequence mutations. Genotyping was performed by matrix-assisted laser desorption by Compass Biotechnology Co., Ltd. (Beijing, China).

Allelic frequencies, genotypic frequencies, polymorphism information content (PIC), and Hardy–Weinberg equilibrium (HWE) were calculated for each locus using PowerMarker Version 3.25. Phased genotypes were partitioned into haplotype blocks using Haploview version 4.2 (Broad Institute, Cambridge, MA, USA). Haploview 4.2 was also used to estimate the LD of all SNPs. The haplotype structure of each buffalo was inferred by the software Phase 2.1.

### 4.3. Association Analysis

Association analysis of *SQLE* polymorphisms and milk production traits was performed with the PROC MIXED procedure of SAS9.4 using the following mixed linear model:

*Y_ijklm_*= *μ* + *G_i_* + *P_j_* + *S_k_* + *F_l_* + *a_m(i)_* + *e_ijklm_*

where *Y_ijklm_* = phenotype observations; *μ* = overall mean; *G_i_* = the fixed-effect of the *i*th genotype or haplotype combination; *P_j_* = the fixed-effect of the *j*th parity (1, 2, 3, 4, and >4); *S_k_* = the fixed-effect of the *k*th season (spring is from March to May, summer is from June to August, autumn is from September to November, and winter is from December to January and February of the following year); *F_l_* = the fixed-effect of the lth farm (four different farms); *a_m(i)_* = the random effects of the *m*th individual buffalo nested within *SQLE* genotype or haplotype combination *i*th; and *e_ijklm_* = the random residual. The least-square means with standard error for multiple comparisons between different genotypes and haplotypes were performed using Tukey correction.

### 4.4. Cell Culture and Transfection

The buffalo mammary gland was obtained from a local slaughterhouse in Wuhan, China. The buffalo mammary epithelial cells (BuMECs) isolation and culture were followed with brief modifications as reference [66]. The BuMECs cells were cultured in DMEM/F12 medium (HyClone, Logan, UT, USA) containing 10% fetal bovine serum (Gibco, Gaithersburg, MD, USA) and 1% penicillin–streptomycin (HyClone, Logan, UT, USA) in a 37 °C incubator with 5% CO_2_. BuMECs cells were cultured into six-well plates overnight. Then, both *SQLE*-siRNA and NC-siRNA were purchased from Shanghai GenePharma Co., Ltd. (Shanghai, China). The cells were transfected with 100 nM of siRNA compared to NC using the Lipofectamine RNAiMAX reagent in Opti-MEM medium (Life Technology, Inc., Carlsbad, CA, USA) instructions. Cells were harvested 48 h after transfection for mRNA and protein expression. Small RNA interference sequences specific to *SQLE* (siSQLE) used in this study are given in Supplementary Table S2.

### 4.5. RNA Extraction and Quantitative Real-Time PCR (qRT-PCR)

Cells were lysed 48 h after transfection to extract total RNA using a kit method (Total RNA Kit I (200); R6834-02; Omega Bio-Tek, Norcross, GA, USA) following the manufacturer’s instructions for subsequent cDNA synthesis. The qRT-PCR was conducted using SYBR Green Master Mix (QIAGEN, Hilden, Germany) to determine the mRNA expression of the target genes using gene-specific primers (Supplementary Table S2). The GAPDH was used as a reference gene, and relative expression was measured using the 2^−ΔΔCt^ method [67].

### 4.6. Western Blotting and Cellular Immunofluorescence

BuMECs transfected with si-SQLE and si-NC in a 6-well plate were placed on ice, washed with PBS, followed by cell lysis using a lysis buffer RIPA (Servicebio, Wuhan, China) augmented with phosphorylase inhibitor (1 mM) and phenylmethanesulphonyl fluoride (PMSF). Proteins were isolated by SDS-PAGE (12%) and subsequently transferred to polyvinylidene difluoride membranes (PVDF; Immobilon-P, Millipore, Burlington, MA, USA) via electrophoresis. Preliminarily, the membranes were incubated with 5% skimmed milk diluted in TBS for 2 h, and subsequently incubated (4 °C, overnight) with these primary antibodies: *SQLE* (1:1000, ER1917-17; HUABIO, Inc., Hangzhou, China), *GAPDH* (1:1000, B1034; Beijing Biodragon Immunotechnologies Co., Ltd., Beijing, China) as a control. Target proteins in each sample were determined using enhanced chemiluminescence (NCI5079; Bio-Rad Laboratories, Hercules, CA, USA). WB images were captured by Gel-pro analyzer version 4 (Media Cybernetics, Rockville, MD, USA), and Image J software (National Institutes of Health, Bethesda, MD, USA) was used to determine bands, respectively. The data were normalized to *GAPDH*. Cellular immunofluorescence was based on a protocol established in our laboratory [68]. Zeiss LSM Image Browser and Adobe Photoshop were used to process the confocal images (Adobe Systems Inc., San Jose, CA, USA).

### 4.7. Cell Counts and Proliferation Assay

The cells were harvested 48 h after transfection of si-SQLE and si-NC for the determination of cell viability or cell numbers. The trypsin-lysed cells were counted using an automated cell counter (BIO-RAD Laboratories, Inc., TC20TM, Hercules, CA, USA). The cell counting kit-8 reagent (Dojindo, Kumamoto, Japan) was supplemented in each well of the experimental group according to the manufacturer’s instructions, and the cells were incubated for 1.5 h at 37 °C with 5% CO_2_. Next, cells were loaded onto a multimode plate reader (PerkinElmer, EnSpire, Waltham, MA, USA) to determine each experimental group’s absorbance at 450 nm.

### 4.8. Cell Cycle and Apoptosis Assay

Cell cycle and apoptosis assay was performed based on a protocol established in our laboratory [69]. Briefly, the cells were pretreated, and the cell cycle was detected by using the cell cycle detection kit (KeyGEN Biotech, Nanjing, China) according to the manufacturer’s instructions. Apoptosis was analyzed by using the Annexin V-FITC/PI Apoptosis Detection Kit (KeyGEN Biotech, Nanjing, China). Finally, according to the manufacturer’s protocol, flow cytometry was used to determine the cell proportions using the FACSVerse Calibur (BD Biosciences, San Jose, CA, USA).

### 4.9. Enzyme-Linked Immunosorbent Assay Were Used to Detect Concentrations of α-, β-, and κ-Casein

Culture media were collected from si-SQLE and si-NC, transfected BuMECs at 48 h, centrifuged at 1000× *g* for 20 min and the supernatant was taken to measure the concentration. ELISA Kit (MLBIO Biotechnology Co., Ltd.; Shanghai, China) was used to measure α-casein (ml572830-1), β-casein (ml572830-2), and κ-casein (ml572830-3) concentrations. The intra- and inter-assay coefficients of variation were less than 10.0% and 10.0% for α-casein, β-casein, and κ-casein.

### 4.10. Triglyceride Content Detection and Oil Red O Staining

A triglyceride enzyme assay kit (Jiancheng Bioengineering Institute, Nanjing, China) was used to determine the triglyceride contents in the BuMECs lysate. The cells were transfected with si-SQLE for 48 h, and 100 μL cell lysate was mixed with the working solution. The absorbance was measured at 510 nm using a microplate reader (PerkinElmer Enspire, Shanghai, China). Cells were stained with oil red O stain kit (G1262; Solarbio, Beijing, China) following the manufacturer’s instructions, operation methods refer to previous studies [70].

### 4.11. Statistical Analysis

The statistical analyses of gene functional studies were conducted with SPSS 19.0 software (SPSS Inc., Chicago, IL, USA) and graphing with GraphPad Prism 8 (GraphPad Software, Inc., La Jolla, CA, USA). The results are expressed as means ± standard error of the mean (Mean ± SEM). Significant differences between the two groups were compared using Student’s *t*-test, and comparisons among multiple groups were performed with a one-way analysis of variance followed by Tukey test. *p*-value < 0.05 was considered statistically significant. All experiments were conducted at least three times.

## 5. Conclusions

This study identified five SNPs and one haplotype block for the *SQLE* gene and found that these mutations were significantly associated with milk composition traits. Furthermore, *SQLE* was found to dramatically affect BuMECs growth (proliferation, cell cycle, and apoptosis) by regulating marker genes (*MYC*, *P21* and *PCNA*) and decreasing the bio-synthesis and secretion of milk triglycerides, lipids, and casein in BuMECs by suppressing their regulatory genes (*RPS6KB1*, *JAK2*, *eIF4E*, and *SREBP1*). These findings prove that the buffalo *SQLE* gene may be a potential candidate gene for marker-assisted selection in the buffalo breeding program.

## Figures and Tables

**Table 1:** Genetic information about five SNPs identified in the buffalo *SQLE* gene.

Snps	Location	Genotypes	Genotype Frequency	Allele	Allele Frequencies	Heterozygosity	PIC
g.14238G > T	intron6	GT	0.244	G	0.870	0.226	0.201
		GG	0.748	T	0.130		
		TT	0.008				
g.16409C > T	intron7	CC	0.207	C	0.462	0.497	0.374
		TT	0.283	T	0.538		
		TC	0.509				
g.18858G > A	Intron7	GG	0.061	G	0.220	0.343	0.284
		AA	0.620	A	0.780		
		GA	0.319				
g.22146C > A	Intron9	CA	0.020	A	0.973	0.053	0.052
		AA	0.963	C	0.027		
		CC	0.017				
g.22834C > T	Intron9	CC	0.518	C	0.510	0.480	0.365
		TT	0.318	T	0.490		
		CT	0.163987				

**Table 2:** Association of *SQLE* gene polymorphisms and milk production traits in Italian Mediterranean buffaloes.

SNPs	Genotype	PM (kg)	MY (kg)	FY (kg)	FP (%)	PY (kg)	PP (%)
g.14238G > T	GG	14.55 ± 0.0.35	2792.28 ± 77.98	214.44 ± 7.00	7.69 ± 0.15	125.74 ± 3.57	4.53 ± 0.04
	GT	14.78 ± 0.38	2855.74 ± 83.00	218.69 ± 7.35	7.68 ± 0.15	128.01 ± 3.82	4.51 ± 0.04
	TT	14.36 ± 1.16	2763.13 ± 249.56	205.24 ± 21.04	7.44 ± 0.42	128.04 ± 11.71	4.65 ± 0.12
	*p*-value	0.60	0.44	0.53	0.81	0.63	0.39
g.16409C > T	CC	15.03 ± 0.24	2896.56 ± 51.01	228.32 ± 4.29	7.89 ± 0.09	131.17 ± 2.40	4.56 ± 0.03
	TC	15.01 ± 0.17	2898.93 ± 35.63	229.30 ± 3.01	7.95 ± 0.06	131.88 ± 1.67	4.57 ± 0.02
	TT	14.85 ± 0.23	2865.60 ± 48.61	228.47 ± 4.09	8.03 ± 0.08	129.69 ± 2.28	4.56 ± 0.02
	*p*-value	0.77	0.81	0.97	0.39	0.68	0.86
g.18858G > A	AA	14.89 ± 0.16	2861.68 ± 35.11	225.47 ± 2.93 ^a^	7.89 ± 0.06	129.75 ± 1.65	4.57 ± 0.02
	AG	15.20 ± 0.20	2929.74 ± 44.07	235.45 ± 3.67 ^b^	8.01 ± 0.07	133.35 ± 2.07	4.57 ± 0.02
	GG	15.23 ± 0.48	2924.20 ± 105.49	233.81 ± 8.73 ^ab^	8.00 ± 0.18	133.14 ± 4.95	4.58 ± 0.05
	*p*-value	0.36	0.37	0.04	0.35	0.28	0.98
g.22146C > A	AA	15.03 ± 0.14	2894.36 ± 30.88	228.22 ± 2.61	7.91 ± 0.05	131.15 ± 1.45	4.56 ± 0.02
	CA	15.46 ± 0.84	3016.19 ± 182.20	234.68 ± 15.38	7.86 ± 0.31	137.92 ± 8.54	4.59 ± 0.09
	CC	15.12 ± 0.80	2924.92 ± 171.91	233.06 ± 14.33	7.93 ± 0.28	132.17 ± 8.09	4.53 ± 0.09
	*p*-value	0.87	0.79	0.87	0.98	0.73	0.90
g.22834C > T	CC	14.70 ± 0.19 ^a^	2821.10 ± 40.20 ^a^	222.50 ± 3.36 ^a^	7.92 ± 0.07	128.07 ± 1.90 ^a^	4.56 ± 0.02
	CT	15.23 ± 0.30 ^ab^	2939.96 ± 64.98 ^ab^	227.13 ± 5.41 ^ab^	7.75 ± 0.11	132.27 ± 3.08 ^ab^	4.53 ± 0.03
	TT	15.31 ± 0.22 ^b^	2951.77 ± 46.75 ^b^	235.56 ± 3.86 ^b^	8.01 ± 0.08	134.53 ± 2.22 ^b^	4.57 ± 0.02
	*p*-value	0.04	0.03	0.02	0.11	0.04	0.46

Note: The values of milk production traits in each genotype are represented as Least squares mean (LSM) ± standard errors (SE). Different superscripts in the same column indicate a significant difference between various groups (^a,b^ *p* < 0.05). MY = milk yield; FY = fat yield; FP = fat percentage; PY = protein yield; PP = protein percentage.

**Table 3:** Association of haplotype combinations of SQLE with milk production traits in Italian Mediterranean buffaloes.

Block	H1H1	H1H3	H2H3	*p*-Value
Sequence	CA/CA	CA/TG	TA/TG	
Number	187	67	53	
Frequencies (%)	56.50	20.24	16.01	
PM (kg)	15.22 ± 0.19	15.07 ± 0.28	14.76 ± 0.17	0.13
MY (kg)	2933.75 ± 41.55	2923.90 ± 60.25	2842.81 ± 37.15	0.15
FY (kg)	235.78 ± 3.44 ^a^	226.97 ± 5.00 ^ab^	225.02 ± 3.09 ^b^	0.03
FP (%)	8.03 ± 0.07 ^a^	7.73 ± 0.10 ^b^	7.94 ± 0.06 ^ab^	0.04
PY (kg)	133.33 ± 1.88 ^a^	131.63 ± 2.73 ^ab^	127.80 ± 1.69 ^b^	0.04
PP (%)	4.57 ± 0.02	4.53 ± 0.03	4.57 ± 0.02	0.44

Note: The values of milk production traits in each genotype are represented as Least squares mean (LSM) ±standard errors (SE). Different superscripts in the same row indicate a significant difference between various groups (^a,b^ *p* < 0.05). PM = peak milk yield; MY = milk yield; FY = fat yield; FP = fat percentage; PY = protein yield; PP = protein percentage.

## Data Availability

The authors confirm that the data supporting the findings of this study are available within the article.

## Supplementary Materials

The following supporting information can be downloaded at: https://www.mdpi.com/article/10.3390/ijms24032436/s1.
