# Supplementary material for: Novel Insight into the Role of Squalene Epoxidase (SQLE) Gene in Determining Milk Production Traits in Buffalo
Source: Int J Mol Sci. 2023 Jan 26;24(3):2436. doi: 10.3390/ijms24032436 (PMC9916492; doi:10.3390/ijms24032436)
Supplement: Supplementary file 1 [file ijms-24-02436-s001.zip › ijms-2137938-supplementary.pdf]

Table S1. Primer information for the buffalo *SQLE* gene.

Primer	Sequence (5’-3’)	Production Size (bp)
1	F: GACTGTACACATACCTATAA	710
	R: TCAAATCTCTGAAGGGATAG	
2	F: GGAAACAAGATTTGGAGACC	703
	R: TTCACCTTACTGTAACCCTG	
3	F: GAACGATAAGGTATGAATAA	620
	R: TGAATCTGCAAGAAGGTGTG	
4	F: CCTTGCTTGATTCACTCCAG	665
	R: TAGGACGAGGTTGCGTGGTG	
5	F: ACACCACGCAACCTCGTCCT	499
	R: GCCTTCTCCCACCAAAGATG	
6	F: ATCTCGGCCTACAAGCGGTG	721
	R: CTCCCCTTTCACGCAATCCA	
7	F: TTGATGTTTGTCTCCCTGTT	599
	R: GTAGCGATAAGACAGCACGA	
8	F: TCGTGCTGTCTTATCGCTAC	435
	R: CTTAATCAAACCCAGTTCGT	
9	F: TCTGACTCTTTGTGACCCTG	693
	R: TTATCCTCTCCTTTGCCTTC	
10	F: GGATCTTAAAGGATGCTTCA	411
	R: ACATCTCTGAGGCTAAGTGG	
11	F: CCACTTAGCCTCAGAGATGT	435
	R: AAAGGACTTATTGACTTGGA	
12	F: TTGTCCCTTGACCAGCAGAT	447
	R: TTTAGAAGCAGGAGGAGTAC	

Table S2. Primer sequences used for qRT-PCR assay and siRNA sequences.

**Genes**	**Primer Sequence (5’-3’)**	**Annealing Temperature (°C)**	**Production Size (bp)**
SQLE	F: GCAGCGCGCATAAGTGTATC<br>R: GAGGAATCAGGCACAGCCTT	60	231
GAPDH	F: AGGTTGTCTCCTGCGACTTC<br>R: ATTCTCAGTGTGGTGGAGGTG	60	263
PCNA	F: GAACCTCACCAGCATGTCCAA<br>R: TTCACCAGAAGGCATCTTTACT	60	220
P21	F: GCCTTCCAGAGGCTCAACTT<br>R: GCTTCGCTATGGTCCTCTCC	60	115
SREBP1	F：AGTAGCAGCGGTGGAAGT<br>R：GCAGCGGCTCTGGATT	58	82
JAK2	F: TGAAGAAAACAGGTAATCAGACTGGA<br>R: AACATTTTCTCGCTCAACAGCA	60	101
RPS6KB1	F: AAATGCTGCTTCTCGTCT<br>R: GTTCTTCCCAGTTAATATGTCT	54	89
eIF4E	F: GTGACCTCGATCGTTTTTGG<br>R: TAGTGGTGGAGCCGCTCTTA	55	264
si-SQLE (1103)	F: CCUAUAUUCUGUCAGCCUUTT<br>R: AAGGCUGACAGAAUAUAGGTT		
si-SQLE (1843)	F: GCACCGCAGUUUAAAGCAATT<br>R: UUGCUUUAAACUGCGGUGCTT		
si-SQLE (1960)	F: CCAAGGAAUUUGAGAGAAUTT<br>R: AUUCUCUCAAAUUCCUUGGTT		
si-SQLE (2031)	F: CCUAGAAGCCUCUCAGAAUTT<br>R: AUUCUGAGAGGCUUCUAGGTT		
